# S145. ANTIPSYCHOTIC DISCONTINUATION IN FIRST EPISODE PSYCHOSIS: [18F]DOPA AND [11C]RACLOPRIDE PET STUDY

**DOI:** 10.1093/schbul/sby018.932

**Published:** 2018-04-01

**Authors:** Euitae Kim

**Affiliations:** Seoul National University Bundang Hospital

## Abstract

**Background:**

Recent meta-analysis revealed that elevated presynaptic striatal dopaminergic function is a robust feature of psychosis like schizophrenia. Considering increased dopaminergic capacity in psychotic disorders, it is not surprising that antipsychotic drugs, which primarily block dopaminergic neurotransmission, are mostly effective in the treatment of psychosis.

However, it remains obscure what would happen to presynaptic dopaminergic function with antipsychotic treatment. This is an important issue addressing whether the current antipsychotic drugs are correcting the primary dopaminergic abnormality or not. In addition, the issue can give a clue regarding the mechanism of relapse in psychotic disorders.

**Methods:**

We measured presynaptic dopamine capacity using [18F]DOPA PET before and after the antipsychotic discontinuation in first episode psychosis. The binding potentials of [11C]raclopride were also measured after the discontinuation. Healthy controls had [18F]DOPA and [11C]raclopride scans at the corresponding date.

First episode psychosis patients were carefully monitored in the aspects of symptomatic aggravations.

**Results:**

The presynaptic dopamine capacity and the density of dopamine receptors showed significant group effect and the interaction between group and time (p<0.005)

**Discussion:**

Dopaminergic function seems to play a critical role in relapse of first episode psychosis.

